# Targeting Selective Autophagy as a Therapeutic Strategy for Viral Infectious Diseases

**DOI:** 10.3389/fmicb.2022.889835

**Published:** 2022-04-28

**Authors:** Yishan Liu, Tao Zhou, Jiajia Hu, Shouheng Jin, Jianfeng Wu, Xiangdong Guan, Yaoxing Wu, Jun Cui

**Affiliations:** ^1^Department of Critical Care Medicine, The First Affiliated Hospital of Sun Yat-sen University, Guangzhou, China; ^2^Ministry of Education (MOE) Key Laboratory of Gene Function and Regulation, State Key Laboratory of Biocontrol, School of Life Sciences, Sun Yat-sen University, Guangzhou, China; ^3^State Key Laboratory of Oncology in South China, Collaborative Innovation Center for Cancer Medicine, Sun Yat-sen University Cancer Center, Guangzhou, China

**Keywords:** selective autophagy, virophagy, antiviral responses, macroautophagy (autophagy), viral infectious diseases

## Abstract

Autophagy is an evolutionarily conserved lysosomal degradation system which can recycle multiple cytoplasmic components under both physiological and stressful conditions. Autophagy could be highly selective to deliver different cargoes or substrates, including protein aggregates, pathogenic proteins or superfluous organelles to lysosome using a series of cargo receptor proteins. During viral invasion, cargo receptors selectively target pathogenic components to autolysosome to defense against infection. However, viruses not only evolve different strategies to counteract and escape selective autophagy, but also utilize selective autophagy to restrict antiviral responses to expedite viral replication. Furthermore, several viruses could activate certain forms of selective autophagy, including mitophagy, lipophagy, aggrephagy, and ferritinophagy, for more effective infection and replication. The complicated relationship between selective autophagy and viral infection indicates that selective autophagy may provide potential therapeutic targets for human infectious diseases. In this review, we will summarize the recent progress on the interplay between selective autophagy and host antiviral defense, aiming to arouse the importance of modulating selective autophagy as future therapies toward viral infectious diseases.

## Introduction

Macroautophagy, hereafter simply referred to as autophagy, is an ancient and highly conserved catabolic degradative process for the elimination of cytoplasmic components through lysosome (Mizushima and Levine, 2010; Mizushima et al., 2011; Nakatogawa, 2020). Cytoplasmic materials destined for autophagic disposal are engulfed by a specialized double-membrane vesicle called autophagosome (Dikic and Elazar, 2018). Autophagosomes then fuse with lysosomes and form autolysosomes through membrane remodeling (Zhao et al., 2021). Autophagy is initially regarded as a starvation-associated bulk degradation process, while it becomes increasingly appreciated that autophagy can target particular substrates through a series of specific cargo receptors. SQSTM1/p62, CALCOCO2/NDP52, OPTN, and NBR1 are well-known cargo receptors that possess both ubiquitin-binding domains (UBDs) and LC3-interacting regions (LIRs) to ensure their capacity to deliver dedicated substrates to autophagosomes (Rogov et al., 2014; Kirkin and Rogov, 2019). Selective autophagy could be classified into multiple subsets according to their degradative substrates, including xenophagy/virophagy for invading intracellular pathogens, mitophagy for mitochondria, aggrephagy for protein aggregates, ER-phagy for endoplasmic reticulum, lipophagy for lipid droplets, and ferritinophagy for ferritin. Based on the subsets of selective autophagy, diverse groups of specific cargo receptors associate with degradative cargoes and deliver their cargoes to autophagy machinery (Table 1).

**TABLE 1:** Selective autophagy cargoes and receptors.

Selective autophagy type	Cargoes	Receptors	References
Xenophagy/Virophagy	Bacterial and viral pathogens, pathogens components	p62	Orvedahl et al., 2010
		OPTN	Wild et al., 2011
		NDP52	Verlhac et al., 2015
		TRIM5a	Mandell et al., 2014
		SCOTIN	Kim N. et al., 2016
		TAX1BP1	Tumbarello et al., 2015
Mitophagy	Damaged or surplus mitochondrial	BNIP3	Zhang and Ney, 2009
		TAX1BP1	Lazarou et al., 2015
		FUNDC1	Liu et al., 2012
		BCL2L13	Murakawa et al., 2019
		AMBRA1	Strappazzon et al., 2020
		PHB2	Yan et al., 2020
		NIX	Yan et al., 2020
		FKBP8	Bhujabal et al., 2017
		NDP52	Lazarou et al., 2015
		OPTN	Wong and Holzbaur, 2014
Aggrephagy	Protein aggregates	TOLLIP	Lu et al., 2014
		p62	Clausen et al., 2010
		NBR1	Kirkin et al., 2009
		OPTN	Korac et al., 2013
Lipophagy	Lipid droplets	ATGL	Dupont et al., 2014
		PNPLA5	Sathyanarayan et al., 2017
		PNPLA8	Kim K. Y. et al., 2016
		PNPLA3	Negoita et al., 2019
Ferritinophagy	Ferritin	NCOA4	Mancias et al., 2014
ER-phagy	ER	FAM134B	Khaminets et al., 2015
		Sec62	Loi et al., 2019
		RTN3	Grumati et al., 2017
		CCPG1	Smith et al., 2018
		ATL3	Chen et al., 2019
		TEX264	Chino et al., 2019
		p62	Yang et al., 2016

As a critical intracellular degradative process, autophagy also participates in cell-intrinsic defense by eliminating invading cytosolic microbes (Deretic et al., 2013; Huang and Brumell, 2014; Choi et al., 2018). Given the powerful cleavage capability, autophagy is generally activated to degrade and dispose of invading viruses during virus infection (Choi et al., 2018). Autophagy also promotes antigen processing to adaptive immune responses against invading viruses in the late stage of infection (Paludan et al., 2005). As counteractions, viruses have also evolved strategies to resist, escape, subvert or even hijack autophagy for their replication (Choi et al., 2018; Viret et al., 2021). For example, viruses could escape from selective autophagic degradation *via* cleaving cargo receptors, restricting autophagy activation and disturbing autophagy pathway. In addition, viruses could promote selective autophagy of key molecules in interferon (IFN) pathway to evade host antiviral innate responses. Viruses could also take advantages of specific types of selective autophagy to enhance viral replication (Choi et al., 2018; Viret et al., 2021; Vo and Choi, 2021).

In this review, we will focus on the involvement of selective autophagy as antiviral defense mechanism and discussed the strategies against selective autophagy by viruses. Additionally, we will discuss whether targeting selective autophagy possesses the potential to develop host-directed therapies as treatment options against viral infectious diseases.

## Functions of Selective Autophagy During Virus Infection

In addition to remove cytoplasmic material such as protein aggregates and damaged or superfluous organelles, selective autophagy has also been reported to mediate autophagic elimination of viral constituents or virions *via* several specific cargo receptors. Selective autophagy contributes to host antiviral responses through directly targeting viral components for degradation as well as facilitating host innate and adaptive immunity.

### Selective Autophagy Targets Viral Components for Degradation

As a part of host immune responses, the fundamental properties of selective autophagy are to sense viral particles or viral components and deliver them to autolysosome for degradation (Liang et al., 2021). Ref(2)P, the p62 ortholog in *Drosophila melanogaster*, was first identified to restrict sigma virus replication through binding its capsid protein (Dru et al., 1993; Wyers et al., 1993), and it could also restrict the replication of vesicular stomatitis virus (VSV) and flavivirus zika virus (ZIKV) (Shelly et al., 2009; Liu et al., 2018). In mammalian, autophagy was first observed to defense virus infection against Sindbis virus (SINV), as defective autophagic pathways enhanced fatal SINV encephalitis in mice (Liang et al., 1998; Mizushima et al., 2001). Subsequently, p62 also exhibited an antiviral role during SINV infection in the central nervous system as it could deliver SINV capsid protein to autophagosome for viral protein clearance (Orvedahl et al., 2010). In addition, p62 was also involved in selective autophagy targeting against double-stranded DNA herpes simplex virus type 1 (HSV-1), adenovirus (AdV) (Orvedahl et al., 2011; Sparrer et al., 2017), positive-sense RNA virus Chikungunya alphavirus (CHIKV), foot-and-mouth disease virus (FMDV) (Berryman et al., 2012; Judith et al., 2013), negative-sense RNA virus measles virus (MeV) (Richetta et al., 2013) and double-stranded RNA virus infectious bursal disease virus (IBDV) (Li Y. et al., 2020). In most case, p62 prefers to target viral capsid or nucleocapsid protein with ubiquitin tags, however, the mechanisms on these preferences still remain unclear (Viret et al., 2021). Besides sensing ubiquitinated capsid proteins, p62 could sense VP1 and VP3 from Seneca Valley virus (SVV) in UBA domain independent manner to inhibit SVV replication (Wen et al., 2021). In addition to ubiquitin tag, Galectin decoration serves as a novel cargo receptor recognition signal (Montespan et al., 2017; Miyakawa et al., 2022). p62 is reported to target hepatitis B virus (HBV) after Galectin-9 decoration (Miyakawa et al., 2022). Reminiscent of the role in targeting viral components, p62 also binds to host factors hijacked by viruses for their replication (Sagnier et al., 2015). In CD4^+^ T lymphocytes, p62 could target human immunodeficiency virus (HIV) transactivator Tat for autophagic degradation, thereby inhibiting HIV replication (Sagnier et al., 2015).

**FIGURE 1:**
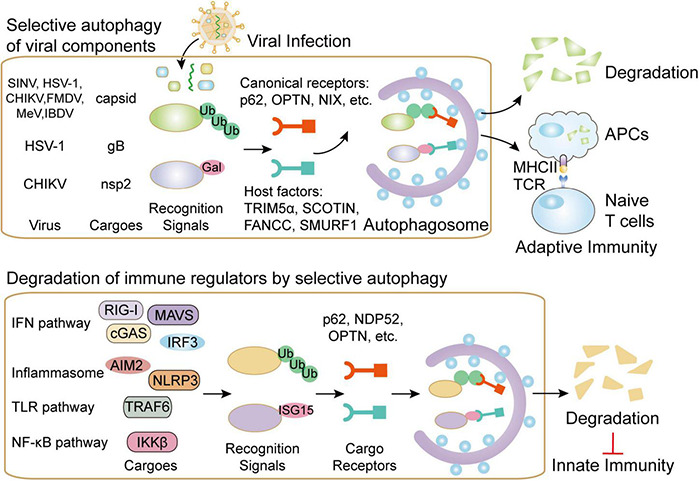
The functions of selective autophagy during viral infection. Selective autophagy targets invading viruses by directly delivering them to autophagosomes for degradation. Several canonical autophagy cargo receptors such as p62 could bind to ubiquitin-coated viral particles or components. Moreover, several host factors, including TRIM5α, SCOTIN, FANCC, and SMURF1, are also reported to assist the transportation of viral components to autophagosomes. In addition to directly targeting viral particles for degradation, selective autophagy also contributes to viral presentation on MHC-II and MHC-I in APCs, thus initiating antiviral adaptive immunity. On the contrary, cargo receptors in selective autophagy pathway could also control autophagic degradation of key molecules in IFN, inflammasome, TLR and NF-κB pathway in order to reduce excessive innate immunity during viral infection.

Along with prototypic roles of p62, several other cargo receptors also participate in autophagic degradation of viral components. OPTN targets HSV-1 tegument protein, VP16, and the fusion glycoprotein, gB, for degradation by selective autophagy in the central nervous system (Ames et al., 2021). NBR can target pararetrovirus cauliflower mosaic virus (CaMV) for autophagic degradation (Hafren et al., 2017). Unlike the other antiviral autophagic receptors, NDP52 seems to promote CHIKV replication through interaction with CHIKV NSP2 in a ubiquitination-dependent manner (Judith et al., 2013). Besides the well-known cargo receptors, several host molecules also act as autophagy receptor-like factors. Fanconi anemia group C protein (FANCC), a factor controlling mitophagy, directly links SINV and HSV-1 capsid with autophagosomes for degradation (Minton, 2016; Sumpter et al., 2016). SMAD ubiquitin regulatory factor 1 (SMURF1) could directly interact with SINV and HSV-1 capsid proteins and mediate their autophagic degradation (Orvedahl et al., 2011; Sumpter et al., 2016). Endoplasmic reticulum (ER) protein SCOTIN restricts hepatitis C virus (HCV) replication by delivering its NS5A to autophagosomes for degradation (Kim N. et al., 2016). Host antiviral factor TRIM5α is also shown to behave as an autophagic receptor by bridging ATG8 factors and HIV capsid factor p24 in cooperation with p62 (Mandell et al., 2014). Hence, host cargo receptors and receptor-like factors direct viral components to autophagosomes for selective degradation, thus contributing to cell-intrinsic defense against viruses (Figure 1).

### The Contribution of Selective Autophagy in Viral Antigen Presentation

Adaptive immune response is an indispensable step for effectively eliminating invaded viruses. Accumulating evidence reveals that autophagy has been extensively involved in antigen presentation (Ireland and Unanue, 2011; Puleston et al., 2014). During viral infection, autophagy is also responsible for MHC-II-meditated antigen presentation (Liang et al., 2021). Epstein–Barr virus (EBV) is presented by MHC-II molecules and then recognized by CD4^+^ T cells, which could be inhibited by blocking of autophagy (Paludan et al., 2005). HIV is also targeted to autophagosome, in which HIV Gag-derived proteins are processed and presented on MHC-II molecules (Kyei et al., 2009). Autophagy also participates in antigen’s internalization and the formation of MHC-I molecules. The latency-associated protein, pUL138 from human cytomegalovirus (HCMV) could be presented by MHC-I through non-conventional transporter associated with antigen processing-independent pathways, which is mediated by autophagy (Tey and Khanna, 2012; Figure 1). Autophagy could process HSV-1 antigens loading on MHC class I molecules, thereby presenting to CD8^+^ cells (English et al., 2009). Despite the incontrovertible roles of autophagy in antigen presentation during viral infection, the participations of cargo receptors in this process have been seldom mentioned. Diverse viral components are selectively presented by MHC-I and MHC-II molecules and then recognized by CD8^+^ and CD4^+^ T cells, however, the contribution of selective autophagy in antigen presentation during infection remains to be illustrated.

## Viral Strategies for Counteracting Selective Autophagy

**FIGURE 2:**
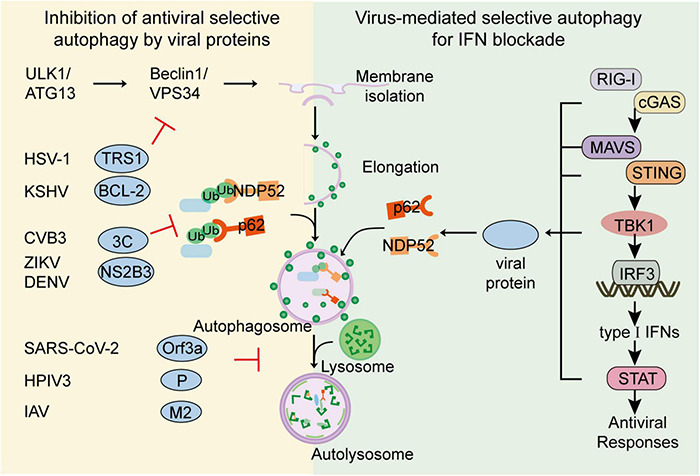
Viral evasion against selective autophagy. To escape the restriction of selective autophagy, viruses have evolved multiple strategies to subvert selective autophagy. Viruses such as CVB3, DENV could directly cleave cargo receptors. Viruses such as HSV-1 or KSHV could inhibit autophagy activation through ATG proteins. Viruses such as SARS-CoV-2 and IAV could restrict the fusion of autophagosome and lysosome for their replication. Additionally, to optimize replication capacity, viruses including H7N9, SARS-CoV-2 and BTV, could also utilize selective autophagy to minimize host antiviral responses. Viruses such as HSV-1, H7N9, and SARS-CoV-2, could utilize cargo receptors to target IFN signal molecules for autophagic degradation.

Although selective autophagy restricts viral replication *via* degrading viral particles and enhancing host immune responses, viruses have evolved multiple strategies to escape or subvert such restrictions. The strategies could be generally summed up as (1) directly cleavage of autophagic cargo receptors; (2) inhibition of autophagy activation through ATG proteins; and (3) restricting autophagy pathway through host inhibitory factors (Yordy and Iwasaki, 2011; Liang et al., 2021). To optimize replication capacity, several viruses also utilize selective autophagy to minimize host antiviral responses (Viret et al., 2021; Figure 2 and Table 2).

**TABLE 2:** Viruses target autophagy receptors to enhance the replication of themself.

Selective autophagy type	Receptor(s)	Viral protein(s)	Mechansim	Outcome
Selective autophagy	p62	CVB3 protease 2A	Cleave p62 and disrupt the selective autophagy	Impair NF-kappaB signaling and promote the proliferation of CVB3
	NDP52	CHIKV nsP2	Anchor the replicative complexes to the trans-golgi network through interacting with NDP52	Promote the proliferation of CHIKV
	NBR1	CVB3 protease 2A and 3C	Cleave NBR1 and disrupt the selective autophagy	Induce increased accumulation of ubiquitin conjugated protein
Virophagy	p62	CVB3 protease 2A	Cleave p62 and disrupt the selective autophagy of CVB3 VP1	Evade host virophagy and promote viral propagation
	NDP52	CVB3 protease 3C	cleave NDP52 and disrupt the selective autophagy of CVB3 VP1	evade host virophagy and promote viral propagation
Mitophagy	NIX	HHV-8 vIRF-1	Promote mitophagy and suppress apoptosis	Promote the proliferation of HHV-8
	IAV PB1-F2	IAV PB1-F2	Act as mitophagy receptors and induce parkin-pink1 independent mitophagy	Suppress IFN responses
	HPIV3 Matrix protein	HPIV3 Matrix protein	Act as mitophagy receptors and induce parkin-pink1 independent mitophagy	Suppress IFN responses
Aggrephagy	p62	CVB3 protease 2A and 3C	Cleave p62/NBR1 and disrupt the selective autophagy	Induce increased accumulation of ubiquitin conjugated protein
	NBR1	CVB3 protease 2A and 3C	Cleave p62/NBR1 and disrupt the selective autophagy	Induce increased accumulation of ubiquitin conjugated protein
	TBC1D5	MCMV M45 and HSV-1 ICP6	Induce the aggregation of RIPK1/NEMO and facilitate the aggrephagy	Promote the proliferation of MCMV and HSV-1
Lipophagy	–	DENV NS4A	Inhibit the acyltransferase activity of AUP1	Promote lipophagy and proliferation of DENV
		DENV NS4B		
		PRRSV	Decrease the expression of NDRG1	Promote lipophagy and proliferation of PRRSV
Ferritinophagy	NCOA4	HCMV pUL38	Restrict the functions of USP24	Promote the proliferation of HCMV
ER-phagy	FAM134B	DENV NS2B3	Cleave FAM134B and subvert ER-phagy	Promote the replication of DENV/WNV/ZIKV
		WNV NS2B3		
		ZIKV NS2B3		

### Viral Escape From Selective Autophagic Degradation by Targeting Cargo Receptors

As the co-evolution between virus and host has taken place over a long period, several viruses have developed various strategies to counteract the restriction initiated by autophagy. The direct strategy employed by viruses to reduce selective autophagy is inducing cargo receptors cleavage. The picornavirus, coxsackievirus B3 (CVB3) utilizes its protease 2A and 2C to cleave cargo receptors p62, NBR and NDP52, which can target CVB3 VP1 capsid protein for degradation (Shi et al., 2013, 2014; Mohamud et al., 2019). SVV 3C^pro^ could target p62 for cleavage, to abolish the capacity of selective autophagy (Wen et al., 2021). Aichi virus 3C protease reduces p62-mediated antiviral response by targeting p62 and LC3 (Kung et al., 2020). Dengue virus (DENV) is shown to promote proteasomal degradation of p62 to restrict autophagic flux (Metz et al., 2015). NS2B3 from DENV and ZIKV can cleave ER-phagy receptor FAM134B to facilitate viral replication (Lennemann and Coyne, 2017).

In addition to cleave cargo receptors, viruses also decrease autophagic flux by inhibiting the activation of ATG proteins. HSV-1 could use its ICP34.5, called TRS1, to restrict autophagy by targeting Beclin-1 (Orvedahl et al., 2007). Kaposi’s sarcoma-associated herpesvirus (KSHV) bears viral homologues of BCL-2, which can compete with cellular BCL-2 for Beclin-1 binding, thereby antagonizing autophagy (Cuconati and White, 2002; Pattingre et al., 2005; Ku et al., 2008). HIV Nef is reported to reduce autophagic maturation by targeting Beclin-1, thus restricting autophagic processing of HIV (Kyei et al., 2009). 2AB protein of Seneca virus A (SVA) is reported to antagonize selective autophagy process by degrading LC3 and inhibit autophagic degradation of viral 3C protein (Sun et al., 2021).

Viruses could also impair selective autophagic degradation by recruiting different host factors. For example, an accessory protein Orf3a of Severe Acute Respiratory Syndrome Coronavirus-2 (SARS-CoV-2) recruits and sequestrates VPS39, the component of host HOPS complex and inhibits the association between HOPS and STX17 or RAB7, thereby preventing the autophagosome-lysosome fusion (Hayn et al., 2021; Koepke et al., 2021; Miao et al., 2021). Similarly, viral phosphoprotein (P) of human parainfluenza virus type 3 (HPIV3) could interact with SNAP29, which restricts the association between SNAP29 and STX17 and inhibits the fusion between autophagosome and lysosome (Ding et al., 2014). Matrix protein 2 of influenza A virus (IAV) is also shown to promote accumulation of autophagosomes by blocking the fusion of autolysosome, thus inhibiting autophagosome degradation and compromising the survival of IAV-infected cells (Gannage et al., 2009; Figure 2).

### Viruses Hijack Selective Autophagy for Interferon Blockade

Type I IFN responses provide the first line of defense against invading viruses. By recognizing cytosolic viral nucleic acid, host RNA sensors including retinoic acid-inducible gene I (RIG-I) -I-like receptors (RLRs) and DNA sensors including cyclic GMP-AMP Synthase (cGAS), initiate signaling cascade through their adaptor proteins, mitochondrial antiviral signaling srotein (MAVS) and stimulator of interferon genes (STING), respectively. Once activated, these adaptors recruit downstream kinase TANK binding kinase 1 (TBK1) to the signalosomes, which subsequently phosphorylate transcription factor interferon regulatory factor 3 (IRF3) to activate type I IFN transcriptions. After binding to a series of cognate receptors, secreted IFN productions induce the expression of various IFN-stimulated genes and initiate antiviral responses (Akira et al., 2006; Loo and Gale, 2011; Schneider et al., 2014). Although signal transmission of type I IFN is critical for antiviral responses, excessive IFN responses would trigger severe immunopathology. Thus, type I IFN signaling requires stringent regulation to ensure effective but not self-injurious antiviral responses. Accumulating evidence reveals that selective autophagy is extensively involved in the regulation of host immunity (Wu and Cui, 2019; Viret et al., 2021). To avoid hyperactivation of IFN responses, several cargo receptors are reported to mediate the selective autophagy of the key signal components at almost each level of IFN signaling pathway, including RIG-I, cGAS, MAVS, STING, TBK1, and IRF3 (Chen et al., 2016; Jin et al., 2017; Du et al., 2018; Wu and Cui, 2019; Wu et al., 2021; Xie et al., 2022). However, recent studies revealed several viruses are capable to subvert selective autophagy to counteract IFN signaling cascade by promoting autophagic degradation of key components in IFN pathway. To attenuate the activation of IFN signaling, porcine circoviruses type 2 (PCV2) could induce phosphorylation of cGAS at S278 *via* activation of PI3K/Akt signaling which abolishes cGAS’s catalytic activity, and subsequently promote the K48-linked ubiquitination of cGAS, which is targeted by HDAC6 and delivered to autolysosome for degradation (Wang Z. et al., 2021). In addition to degrade viral receptors, several viruses promote the autophagic degradation of adaptor proteins in IFN pathway to block signal amplification. PB1 protein of influenza A virus subtype H7N9 (H7N9) is shown to recruit host E3 ligase RNF5 to induce K27-linked ubiquitination of MAVS at its Lys362 and Lys461, which will subsequently be recognized by cargo receptor NBR1 and lead to its autophagic degradation. Consequently, H7N9 PB1 protein-mediated degradation of MAVS leads to lessened RIG-I-MAVS IFN signaling and enhanced H7N9 infection (Zeng et al., 2021). SARS-CoV-2 helicase NSP13 can promote the autophagic degradation of TBK1, the central kinase in IFN pathway in cooperation with cargo receptor p62, thus in turn inhibiting IFN productions (Sui et al., 2022). Besides restricting the generation of type I IFN, viruses may also enforce selective autophagy to further soften IFN signal transduction. Bluetongue virus (BTV) NS3 is responsible to recruit host E3 ligase to ubiquitinate transcription factor STAT2 and induce its autophagic degradation for IFN blockade (Avia et al., 2019). Apart from cutting off IFN antiviral responses, viruses can compel cargo receptors to degrade host factors directly to enhance replication. HIV gp120 interacts with stathmin and trigger its autophagic degradation to enhance mucosal permeability for viral infection (Xie et al., 2020), and stress-induced corticosterone can induce autophagic degradation of PML, a famous antiviral ISGs, to increase susceptibility to HSV-1 in brain (Maarifi et al., 2014; Li W. et al., 2020). Collectively, virus-utilized selective autophagy may be involved in every step of IFN antiviral process from IFN production to antiviral factors, thus benefiting viral infection (Figure 2).

## Viral Modulation of Organelles-Specific Selective Autophagy During Virus Infection

According to the category of degradative substrates, autophagy can be divided into several specific subsets, including mitophagy for mitochondria, aggrephagy for aggregated proteins, nucleophagy for nucleus, ferritinophagy for ferritin, and lipophagy for lipid droplets (Stolz et al., 2014; Kirkin, 2020). In order to ensure efficient replication, viruses employ and even activate certain forms of organelles-specific autophagy (Vo and Choi, 2021, Table 2).

### Mitophagy

Mitophagy, the most-studied organelle-specific autophagy, is the selective clearance of damaged or surplus mitochondria process, which acts as an important role in mitochondrial quality and quantity control. Hypoxia, mitochondrial depolarization and viral infection can induce mitophagy. In mammalian cells, mitophagy can be processed in E3 ligase parkin RBR E3 ubiquitin protein ligase (PRKN)-dependent and independent way (Kim et al., 2007; Youle and Narendra, 2011). In PRKN-dependent mitophagy, PINK1 accumulates on the outer membrane of mitochondria and recruits PRKN, which can link more ubiquitin chains to mitochondria to promote the engulfment of mitochondria by phagophores to form mitophagosomes (Kondapalli et al., 2012). In PRKN-independent mitophagy, several mitophagy receptors located on the outer membrane of mitochondria, including FUNDC1, NIX, BCL2L13, and BNIP3, can interact with LC3 through LIR motif and promote the engulfment of mitochondria (Schweers et al., 2007; Zhang and Ney, 2009; Liu et al., 2012; Murakawa et al., 2019). After the engulfment of mitochondria, the phagophores form mature mitophagosomes, then fuse with lysosomes and degrade the mitochondria (Youle and Narendra, 2011).

During viral infection, portions of viral proteins can influence the process of mitophagy to shape an environment for viral replication (Zhang L. et al., 2018). The activation of RIG-I-mediated IFN antiviral responses depends on the oligomerization of MAVS, which located on mitochondrial outer membrane, therefore mitophagy suppresses the activation of IFN responses to a certain extent (Jacobs and Coyne, 2013). Accordingly, RNA viruses evolved mechanisms to achieve immune evasion by inhibiting the activation of IFN pathway through promoting mitophagy. For example, HPIV3 matrix protein and IAV F2 protein can interact with mitochondrial elongation factor TUFM and LC3 to induce mitophagy, which inhibits the activation of IFN responses (Ding et al., 2017; Wang R. et al., 2021). NS1 of influenza virus could disrupt activity of mitochondria and enhance mitophagy *via* host ULK1 and BNIP3 (Lee et al., 2021). Moreover, DNA virus human herpes virus 8 (HHV-8) can also induce mitophagy through the interaction between viral protein vIRF-1 and mitophagy receptor NIX, which can promote cell survival *via* reducing mitochondria-induced cytotoxicity (Vo et al., 2019). HHV-8 vIRF-1 is also noted to promote mitophagy and suppress caspase-8-mediated apoptosis through interacting with TUFM, thus promoting the survival of lytic infected cells (Choi et al., 2022).

Apoptosis is a classic and well-studied programmed cell death modality. It has been widely reported that the apoptosis of viral infected cells is one of the crucial immune responses against viral infection (Kvansakul, 2017). The initiation of apoptosis is also dependent on mitochondria, which could be degraded and inhibited by mitophagy to promote viral replication (Galluzzi et al., 2008). Indeed, several viruses have developed strategies to prevent apoptosis *via* inducing mitophagy. Classic swine fever virus (CSFV) NS3 can interact with lactate dehydrogenase B (LDHB) and decrease the expression of LDHB, which leads to the activation of mitophagy and inhibition of apoptosis, thus enhancing the replication of CSFV (Fan et al., 2021). As the reinforcement of mitophagy in promoting viral propagation, targeting mitophagy may become a potential therapeutic treatment against certain viral infectious diseases.

### Aggrephagy

Protein aggregation is generated and accumulated as a result of improper folding or misfolding due to mutation, incomplete translation, abnormal post translational modification and oxidative stress (Dobson, 2003). There are two major protein quality control mechanisms including molecular chaperones and ubiquitin-proteasome system (UPS) (Kaufman et al., 2002). Chaperones are proteins that assist in the assembly and folding of proteins, which can recognize and repair misfolded proteins (Bukau and Horwich, 1998). UPS is another major protein degradation system, which can recognize those ubiquitinated soluble misfolded proteins (Kaufman et al., 2002). Protein aggregation can also be recognized and eliminated by selective autophagy, which called aggrephagy (Lamark and Johansen, 2012). The initiation of aggrephagy is mediated by several autophagy receptors including Toll interacting protein (TOLLIP), p62/SQSTM1, NBR1, and OPTN (Kirkin et al., 2009; Clausen et al., 2010; Korac et al., 2013; Muscolino et al., 2020). It has been reported that viruses can utilize aggrephagy to escape from immune surveillance. HCMV M45 protein can induce the aggregation of host proteins receptor-interacting serine/threonine-protein kinase 1 (RIPK1) and NF-kappaB essential modulator (NEMO) (Muscolino et al., 2020), which functions in the activation of NF-κB signaling pathway and apoptosis/necroptosis pathway through its IPAM motif (Kondylis et al., 2017). The aggregation of RIPK1 and NEMO would abolish their functions and induces the selective degradation, thereby resulting in the inhibition of premature cell death and viral replication (Muscolino et al., 2020). In addition, HSV-1 ICP6 protein is demonstrated to induce RIPK1 aggregation and degradation in a similar pattern as HCMV M45 does, suggesting the pro-viral role of aggrephagy in virus infection (Muscolino et al., 2020).

### Lipophagy

Lipid droplets (LDs), organelles with a monolayer phospholipid membrane, are the main storage site of intracellular neutral lipids (Olzmann and Carvalho, 2019). Multiple lipids including cholesterol esters and triglycerides are stored within LDs. The reservation of lipids is indispensable for the energy metabolism and lipid homeostasis, however, excessive accumulation of lipid can lead to lipotoxicity and cell damage, therefore, the biogenesis and degradation of LDs are required to be an issue of dynamic equilibrium (Jones, 2016). Lipophagy is a subset of selective autophagy for degradation of LDs. Cells can regulate lipid metabolism through selectively degrading triglycerides stored in lipid droplets *via* lipophagy to free fatty acids by the lysosomal acid lipase (LAL) and generating ATP (Singh and Cuervo, 2012; Ward et al., 2016). Several cargo receptors are involved in lipophagy (Shin, 2020). Adipose triglyceride lipase (ATGL) can bind to LDs and interact with LC3 *via* LIR motif, straightly inducing lipophagy (Sathyanarayan et al., 2017). Small G protein Rab family and lipase located on LDs surface such as patatin-like phospholipase domain-containing enzyme 5 (PNPLA5) and patatin-like phospholipase domain-containing enzyme 8 (PNPLA8) also contribute to the recognition and engulfment process of lipophagy (Shin, 2020). Considering the momentous role of lipophagy in lipid metabolism regulation, and given the close relationship between lipid and the regulation of immune system (Yan and Horng, 2020), it is necessary to comprehensively explore the association between lipophagy and viral infection.

The most studied viruses associated with lipophagy are flaviviruses. DENV NS4A and NS4B can interact with LDs surface protein AUP1 and inhibit the acyltransferase activity of AUP1, thus facilitating lipophagy and promoting the generation of ATP for virus replication (Heaton and Randall, 2010; Zhang J. et al., 2018). It is noteworthy that DENV infection activates lipophagy in an AMP-activated protein kinase (AMPK)-dependent manner, which is unnecessary in autophagy activation (Jordan and Randall, 2017). Meanwhile, NS5 of HCV and capsid protein of DENV can accumulate on the surface of LDs, which can promote the assembly of virions (Samsa et al., 2009; Filipe and McLauchlan, 2015). Thus, the association between virus-induced lipophagy and LDs accumulation requires further investigations. In addition to flaviviruses, porcine reproductive and respiratory syndrome virus (PRRSV) infection can decrease the expression of N-Myc downstream-regulated gene 1 (NDRG1) to promote lipophagy and therefore enhance virus replication (Wang et al., 2019). Future studies should focus on how to inhibit the viral-advantageous lipophagy, while attenuating the lipotoxicity caused by the LDs accumulation is a potential therapeutic goal.

### Ferritinophagy

Ferritinophagy is another subset of selective autophagy, refer to the selective autophagic degradation of ferritin, an iron-sequestering protein complex (Kaur and Debnath, 2015). Iron is an important nutrient that can affect the activity of iron-dependent enzymes and then influence a variety of different biochemical reaction processes in cells (Cassat and Skaar, 2013). Excessive free iron will induce the accumulation of lipid peroxidation product and cause the oxidative stress and cytotoxicity (Pantopoulos et al., 2012). The balance of intracellular iron metabolism, an essential cellular process for normal cell physiology, which is controlled by ferritinophagy through selective degradation of ferritin (Kaur and Debnath, 2015). NCOA4, the specific ferritinophagy receptor, can interact with FTH1, a subunit of ferritin complex and autophagy protein LC3, hence promotes the engulfment of ferritin complex by phagophores. Upon fusion with lysosomes, ferritin complexes are degraded, thereby resulting in the release of free iron (Mancias et al., 2014). Ferritinophagy mediates the degradation of ferritin and the release of free iron, which contributes ferroptosis and ROS generation *via* fenton reaction (Ajoolabady et al., 2021). Although it has been reported that viruses can utilize ferroptosis to spread infection (Ashida et al., 2011), ferroptosis can play a positive role in antiviral immune response (Jorgensen et al., 2017). In order to ensure replication, viruses have developed several ways to inhibit ferroptosis caused by ferritinophagy. HCMV pUL38 can interact with host deubiquitinase USP24 which can stabilize ferritinophagy receptor NCOA4. By interacting with USP24, pUL38 can restrict the functions of USP24 and inhibit ferroptosis, thus promoting the proliferation of HCMV (Sun et al., 2018). Moreover, the V protein of human parainfluenza virus 2 (hPIV2) can bind to NOCA4 and ferritin subunit FTH1, thus competitively suppressing ferritinophagy and ferroptosis, resulting in efficient amplification of hPIV2 (Ohta et al., 2021).

## Antiviral Therapy by Targeting Selective Autophagy

As discussed above, selective autophagy plays a vital role during viral infection, which leads us to explore the antiviral interventions by targeting selective autophagy. However, as the double-edged sword effects of autophagy during viral infection, it remains to be challenged to distinguish whether autophagy needs to be upregulated or restricted. Additionally, accumulating evidence reveals that non-specific induction of autophagy as antiviral therapies leads to unexpected side effects with limiting effects on viral evasion. Thus, it is essential for therapy development against infectious diseases by targeting autophagy in a more selective and controlled way with specific degradative cargoes (Table 3).

**TABLE 3:** Antiviral therapy targeting autophagy/selective autophagy.

Target	Product name	Description	Targeted viruses	Effect
Macro-autophagy	Sirolimus/Rapamycin	The serine-threonine kinase mTOR inhibitor	SARS-CoV-2, MERS, H1N1	Autophagy activator
	Metformin	Increasing insulin sensitivity and autophagy	SARS-CoV-2, HCV, HBV, HIV	Autophagy activator
	Ivermectin	The AKT phosphorylation inhibitor	SARS-CoV-2	Autophagy activator
	Wortmannin	Selective PI3K inhibitor	ZIKV	Autophagy inhibitor
	CSC27	Selective mTORC2 blocker	EBV	Autophagy activator
	Corticosteroids	LC3 recruitment blocker	H1N1, SARS, MERS, SARS-CoV-2	Autophagy inhibitor
CMA	Monoclonal antibody	A monoclonal antibody against HSC70	Rotavirus	Autophagy inhibitor
	Oxymatrine	Selective HSC70 inhibitor	HBV, ADV, ETV	Autophagy inhibitor
	VER-155008	Competitive HSP70 inhibitor	IBDV	Autophagy inhibitor
Lysosome	CQ/HCQ	Antimalarial agent/Heme polymerase inhibitor	ZIKV, CHIKV, HIV, HCV, SARS-CoV-2	Autophagy inhibitors
	Moringa A	TFEB inhibitor	IAV	Autophagy inhibitor
	Trehalose	Naturally MTOR-independent autophagy inducers	HIV	Autophagy activator
Selective autophagy	Valinomycin	Cyclodepsipeptide antibiotic	SARS-CoV, MERS-CoV, SARS-CoV-2	Mitophagy activator
	Agonists of the sigma-1 receptor	Mitophagy activator	HCV, SARS-CoV-2	Mitophagy activator

### Antiviral Therapeutic Strategies Toward Autophagy

Macroautophagy is the best-characterized form of autophagy, involving a specialized double-membrane vesicle known as the autophagosome which delivers virus-derived antigens for presentation to T lymphocytes. The outbreak of the novel coronavirus SARS-CoV-2 has infected thousands of people since the end of 2019 and caused worldwide social and economic disruption. Sirolimus (also known as rapamycin) has been shown to be a possible respiratory syndrome inhibitor against virus such as SARS-CoV-2, MERS as well as influenza A virus subtype H1N1 (H1N1) (Keating et al., 2013; Kindrachuk et al., 2015; Husain and Byrareddy, 2020). Beyond that, metformin which has been well-known for its pleiotropic effects also has a possible role in combating SARS-CoV-2 as well as HCV, HBV or HIV through increasing insulin sensitivity and autophagy (Chen et al., 2018; Sharma et al., 2020). As an autophagy inducer, ivermectin is likely to have a significant impact on SARS-CoV-2 by inhibiting the AKT phosphorylation, which has been demonstrated in numerous clinical trials (Bryant et al., 2021). It has been reported that pharmacological inhibition of autophagy using wortmannin—an inhibitor of autophagy by blocking PI3K—is associated to the reduction of ZIKV replication in human umbilical vein endothelial cells (Peng et al., 2018).

Apart from traditional mTOR inhibitors, further investigations had brought the use of autophagy activators to the forefront. A representative compound, CSC27 (manassantin B), abrogates EBV lytic replication by specifically blocking mTORC2-mediated phosphorylation and suppresses the expression of EBV immediate-early gene BZLF1, providing a candidate compound for further development of a new drug with minimal cytotoxicity to treat EBV-associated diseases (Wang Q. et al., 2020). Reminiscent of the role in targeting immune system, corticosteroids is known to inhibit autophagy by blocking LC3 recruitment (Kyrmizi et al., 2013), and its clinical roles in viral infection have been investigated in H1N1, SARS, MERS and SARS-CoV-2 (Auyeung et al., 2005; Arabi et al., 2018; Lipworth et al., 2020).

### Antiviral Therapeutic Strategies Targeting Chaperon-Mediated Autophagy

Chaperon-mediated autophagy (CMA) directly delivers substrate proteins into the lysosome lumen using the cytosolic chaperone HSC70 and the lysosomal receptor LAMP-2A for degradation. Recent studies have shown that HSC70 can be a promising target for antivirals. A monoclonal antibody against HSC70 could block the infectivity of Rotavirus (Pérez-Vargas et al., 2006). Similarly, a selective HSC70 inhibitor, oxymatrine, suppresses *de novo* synthesis of both the wild-type HBV strain and strains resistant to 3TC, ADV, and ETV, presumably through destabilization of *Hsc70* mRNA (Gao et al., 2011). As an ATP analog, adenosine derivative compound VER-155008 acts as a competitive inhibitor to bind the HSP70 family and induces the conformational changes of HSC70, making it impossible for HSC70 to interact with IBDV VP2 protein and thus inhibiting virus replication (Chen et al., 2020).

### Targeting Lysosomal Function

Chloroquine (CQ) and hydroxychloroquine (HCQ) have been used as antimalarial agents for many years. Based on its effects on the annulation of endosomal/lysosomal acidification, CQ showed its antiviral effects against ZIKV, CHIKV, and HIV (Al-Bari, 2017; Peng et al., 2018). A randomized, triple-blind, placebo-controlled pilot trial reports that treatment with chloroquine reduces the HCV viral RNA levels in non-responders to other viral treatments (Peymani et al., 2016). In 2020, CQ and HCQ were approved for the U.S. Food and Drug Administration (FDA) for the treatment of infected patients with the coronavirus SARS-CoV-2 (Martinez et al., 2020), and its effect had been proved in abundant clinical trials and researches *in vitro* (Wang M. et al., 2020; Yao et al., 2020). However, the potential adverse effect of CQ and HCQ cannot be ignored since they may make acute kidney injury (AKI) and other organ failures worse (Edelstein et al., 2020). Recent years, more efficient small molecules are under development for instead of CQ and HCQ. ROC-325, a dimeric small molecule containing core motifs of HCQ and lucanthone, has approximately 10-fold greater anticancer activity and autophagic inhibition than HCQ (Jones et al., 2019), and may go into a phase I clinical trial in the near future.

Transcription factor EB (TFEB) is a key transcription molecule that regulates autophagy at the transcriptional level. Given the modulation of TFEB is known to regulate lysosome biogenesis and autophagy, inhibiting TFEB to block the lysosome production may be a promising strategy for host against viruses. *Moringa* A, a new compound from *Moringa oleifera* seeds, can inhibit the expression and nuclear transfer of TFEB and weaken the autophagy in infected cells, which could be an important antiviral mechanism (Xiong et al., 2021). Trehalose can activate PIKfyve leading to TFEB nuclear translocation in MCOLN1-dependent manner to induce autophagy. Remarkably, trehalose significantly reduced HIV-p24 levels in *ex-vivo*-infected PBMCs or PBMCs from therapy naïve HIV patients (Sharma et al., 2021).

### Antiviral Therapy Targeting Selective Autophagy

Two years of scientific exploration has attested that autophagy plays an essential role in SARS-CoV-2-mediated COVID-19, and modulating autophagy by using various drugs that act on potential targets of the virus seems to be an undoubtedly promising therapeutic solution for SARS-COV2 infection and replication. Preclinical studies have suggested repurposing a few FDA-approved drugs to treat COVID-19, and almost half of these well-recognized medications are thought to be inhibitors of the pathways involved in autophagy (Mijaljica and Klionsky, 2020). As mentioned above, treatments toward macroautophagy have a splendid contribution to anti-coronaviral therapy. However, clinical trials showed that the potential side effects of autophagy inhibitors destroyed more than virus. CQ cannot be regarded as an ideal option as it may cause simultaneous tissue damage when the therapeutic goal was to decelerate replication (Edelstein et al., 2020), while corticosteroid-treated patients were also more likely to develop myocardial injury (15.6 vs. 10.4%, *P* = 0.041), acute liver injury (18.3 vs. 9.9%, *P* = 0.001), and shock (22.0 vs. 12.6%, *P* < 0.001) compared with controls during hospitalization (Liu et al., 2020). Thus, it desires an urgently needed solution for antiviral therapy to look for more specific drug targets with limiting influences on normal physiological processes, in which selective autophagy may play a considerable role. Enhancing p62-mediated selective autophagy, as we mentioned above, may accelerate virus protein clearance of SINV, HSV-1, and AdV (Viret et al., 2021). Stabilization of novel types of autophagosomes involving in the presentation of various phagocytosed antigens by MHC class I and II molecules may help us fighting with virus infection through coordinating adaptive immunity. When viruses apply selective autophagy for IFN blockade, autophagy inhibitors specifically targeting cargo receptors which are involved in the autophagic degradation of key components in IFN pathway could reverse IFN level and enhance antiviral response. However, it is necessary to foster and extend robust research involving all the pros and cons of selective autophagy therapy before it eventually becomes a priority.

Targeting organelle-specific selective autophagy might also provide a potential therapeutic strategy against infectious diseases. During infection, viruses altered mitochondrial dynamics in order to modulate mitochondria-mediated antiviral immune responses *via* the alteration of mitochondrial events such as mitophagy to facilitate their proliferation (Singh et al., 2021). Valinomycin stimulates mitophagy by loss of MMP, which is shown to inhibit the replication of coronaviruses (Wu et al., 2004; Klein et al., 2011; Zhang et al., 2020). Agonists of the sigma-1 receptor could provide protection of the mitochondria by activating mitophagy to remove damaged and leaking mitochondria. Agonists of the sigma-1 receptor are also shown to prevent cell death in response to HCV and SARS-CoV-2 infection by inducing autophagy (Friesland et al., 2013; Brimson et al., 2021).

## Conclusion

The involvements of selective autophagy have been largely established during viral infection. A set of cargo receptors are involved in autophagic clearance viral factors, thus restricting viral replication. The presentation of viral antigens to active host adaptive immunity also requires the participation of autophagy, and the contributions of autophagic receptors still need to be evaluated. On the contrary, viruses have also evolved strategies to counteract selective autophagy, through degrading cargo receptors or inhibiting receptor’s activities through autophagic flux or other host factors. Autophagic receptors-mediated infection-related calibration of IFN signaling is also disturbed by viruses, which could lead to the reduction of IFN antiviral responses. Moreover, several viruses can induce and utilize certain forms of selective autophagy, including mitophagy, aggrephagy, and lipophagy, to benefit viral replication.

Considering close relationship between selective autophagy and viral replication, antiviral interventions targeting selective autophagy are still at a very early stage. How to effectively modulate autophagy, even selective autophagy, remains to be a long-standing challenge in the implementation of autophagy-based therapies. Given the devastating consequences of the current COVID-19 pandemic and its long-lasting impact on all of us, viral infection and specific treatments toward it are with no doubt of high concern. Researchers have attested that cellular degradation mechanism known as autophagy might be a beneficial approach to fight numerous viruses by using inhibitors or enhancers. As autophagy acts as a double-edged sword during viral invasion, potent regulators based on overall induction of autophagy may have the potential to cause unexpected side effects such as shock, superinfection or even life-threaten multiple organ failure. Instead, more selective approaches that allow only particular autophagic pathways or steps to be targeted has come to the center stage of antiviral therapy. Further robust and interdisciplinary investigations are in great need to looking for more appropriate therapies based on specific autophagy modulation, so that they can be implemented to treat highly contagious virus in the future.

## Author Contributions

JC and YW initiated and designed the project. JW and XG provided suggestions. JH and SJ provided technical assistance. JH draw the schematic diagram. YW, TZ, and JH collect the references. JC, XG, YW, YL, and TZ wrote the manuscript. All authors contributed to the article and approved the submitted version.

## Conflict of Interest

The authors declare that the research was conducted in the absence of any commercial or financial relationships that could be construed as a potential conflict of interest.

## Publisher’s Note

All claims expressed in this article are solely those of the authors and do not necessarily represent those of their affiliated organizations, or those of the publisher, the editors and the reviewers. Any product that may be evaluated in this article, or claim that may be made by its manufacturer, is not guaranteed or endorsed by the publisher.

## Abbreviations

UBD: Ubiquitin-binding domain
LIR: LC3-interacting region
IFN: Interferon
VSV: Vesicular stomatitis virus
ZIKV: Zika virus
SINV: Sindbis virus
HSV-1: Herpes simplex virus type 1
AdV: Adenovirus
CHIKV: Chikungunya alphavirus
FMDV: Foot-and-mouth disease virus
MeV: Measles virus
IBDV: Infectious bursal disease virus
SVV: Seneca valley virus
UBA: Ubiquitin associated domain
HBV: Hepatitis B virus
HIV: Human immunodeficiency virus
CaMV: Cauliflower mosaic virus
FANCC: Fanconi anemia group C protein
SMURF1: SMAD ubiquitin regulatory factor 1
ER: Endoplasmic reticulum
HCV: Hepatitis C virus
MHC: Major Histocompatibility complex
EBV: Epstein-Barr virus
HCMV: Human cytomegalovirus
TAP: Transporters associated with antigen processing
CVB3: Coxsackievirus
AIV: Aichi virus
DENV: Dengue virus
ATGs: Autophagy-related genes
KSHV: Kaposi’s sarcoma-associated herpesvirus
SVA: Seneca virus A
SARS-CoV-2: Severe Acute Respiratory Syndrome Coronavirus-2
HOPS: Homotypic fusion and protein sorting
HPIV3: Human Parainfluenza virus 3
IAV: Influenza A virus
RLRs: Retinoic acid-inducible gene I (RIG-I) -I-like receptors
cGAS: cyclic GMP-AMP Synthase
MAVS: Mitochondrial Antiviral Signaling Protein
STING: Stimulator of Interferon Genes
TBK1: TANK Binding Kinase 1
IRF3: Interferon Regulatory Factor 3
PCV2: Porcine circoviruses type 2
H7N9: influenza A virus subtype H7N9
BTV: Bluetongue virus
ISGs: Interferon-stimulated genes
PRKN: Parkin RBR E3 Ubiquitin Protein Ligase
HHV-8: Human herpes virus 8
CSFV: Classic swine fever virus
UPS: Ubiquitin-proteasome system
TOLLIP: Toll Interacting Protein
RIPK1: Receptor-interacting serine/threonine-protein kinase 1
IPAM: Induced protein aggregation motif
LDs: Lipid droplets
LAL: Lysosomal acid lipase
ATGL: Adipose triglyceride lipase
PRRSV: Porcine reproductive and respiratory syndrome virus
ROS: Reactive oxygen species
HPIV2: Human parainfluenza virus 2
MERS: Middle east respiratory syndrome coronavirus
H1N1: influenza A virus subtype H1N1
CMA: Chaperon-mediated autophagy
3TC: Lamivudine
ADV: Adefovir
ETV: Entecavir
CQ: Chloroquine
HCQ: Hydroxychloroquine
FDA: U.S food and drug administration
AKI: Acute kidney injury
TFEB: Transcription factor EB
PBMCs: Peripheral blood mononuclear cells
MMP: Mitochondrial membrane potential.
